# Survey on *Kudoa thyrsites* in *Sardina pilchardus* From the Central Tyrrhenian Sea

**DOI:** 10.1111/jfd.14123

**Published:** 2025-03-24

**Authors:** Paolo Cipriani, Marialetizia Palomba, Luca Ruggiero, Stig Mæle, Lucilla Giulietti

**Affiliations:** ^1^ Department of Public Health and Infectious Diseases, Section of Parasitology Sapienza University of Rome Italy; ^2^ Institute of Marine Research (IMR) Bergen Norway; ^3^ Department of Ecological and Biological Sciences (DEB) Tuscia University Viterbo Italy; ^4^ Fish Auditing Coop Marinai e Caratisti Scrl Civitavecchia (Rome) Italy

**Keywords:** Kudoa thyrsites, Mediterranean Sea, myoliquefaction, *Sardina pilchardus*, soft flesh

## 1 Introduction

*Kudoa* species are microscopic endoparasites of marine and estuarine fish with a global distribution (reviewed by Levsen 2015). The life cycle of *Kudoa* remains unknown, but it is believed to follow a two‐host cycle involving fish and an alternate host, likely a polychaete (Eszterbauer et al. 2015, Rangel et al. 2016). Some species can affect the quality of fish fillets by producing macroscopic cysts or post‐mortem myoliquefaction, commonly referred to as ‘soft flesh’ condition (see reviews by Levsen 2015; Moran, Whitaker and Kent 1999). *Kudoa thyrsites* is a well‐known ‘soft flesh’‐inducing species that infects the skeletal muscle of several teleost fishes worldwide, including commercially important wild species such as Atlantic mackerel ( *Scomber scombrus* ), Atlantic cod ( *Gadus morhua* ), mahi mahi (*Coryphena hippurus*) and small pelagics (reviewed by Henning, Hoffman, and Manley 2013, and Levsen 2015; Giulietti et al. 2024a, 2024b, 2024c). ‘Soft flesh’ develops only in heavily infected fish with high parasite densities (Giulietti et al. 2022; St‐Hilaire et al. 1997a).

European sardine ( *Sardina pilchardus* ) is a clupeid pelagic fish distributed across the Eastern Atlantic Ocean from Senegal to the North Sea and throughout the Mediterranean Sea (Jemaa et al. 2015; Parrish, Serra and Grant 1989; Silva et al. 2015). Sardines are among the most commercially important species in the Mediterranean, caught using purse seines and pelagic trawls, and play a significant role in traditional cuisine (FAO 2023). In the Northeast (NE) Atlantic, cases of ‘soft flesh’ in sardines have been linked to *K. thyrsites* infections. The first documented case occurred in the 1990s in Portuguese waters, causing significant economic losses to the canning industry (Menezes et al. 1990). Subsequent infections with *K. thyrsites* and associated ‘soft flesh’ have been reported in several sardine stocks, including the Iberian stock from Portugal and northern Spain, and the Northern African stock from Moroccan Atlantic waters (Cruz, Silva, and Saraiva 2011; Iglesias et al. 2022; Gilman and Eiras 1998; Giulietti et al. 2024b, 2024c). In contrast, little is known about *K. thyrsites* infections in sardines from the Mediterranean Sea. *K. thyrsites* has been identified as the causative agent of ‘soft flesh’ in two specimens of silver scabbardfish ( *Lepidopus caudatus* ) from the Alboran Sea (Giulietti et al. 2019) and in one specimen of Atlantic bonito ( *Sarda sarda* ) from the western Mediterranean (Panebianco et al. 2024). Moreover, a case of ‘soft flesh’ caused by an unidentified *Kudoa* species was reported in swordfish ( *Xiphias gladius* ) caught off Sicily (Gaglio et al. 2010). Other ‘soft flesh’‐inducing *Kudoa* species reported in the Mediterranean include *K. camarguensis*, identified as causing muscle softening in two goby fish ( *Pomatoschistus minutus* and *P. microps* ) from the Rhône River delta in southern France (Pampoulie et al. 1999).

The aim of this research was to investigate the occurrence of *K. thyrsites* in sardines from the Tyrrhenian Sea in the central Mediterranean.

## 2 Materials and Methods

A total of 1200 European sardine individuals, caught off Civitavecchia, in the Tyrrhenian Sea (central Mediterranean Sea, FAO area 37.1.2), were obtained at harbour landings straight from fishing boats. The samples were obtained in two batches: 670 fish in August 2023 and 530 in October 2024. Fish were measured (total length, TL; mm) and weighed (total weight, TW; g). To investigate *K. thyrsites* infection in sardine, a two‐step approach was employed: (i) we first used the occurrence of ‘soft flesh’ as an indicator of infection, following the method described by Giulietti et al. (2022, 2024b) and (ii) we then applied molecular techniques to a subsample of sardines to screen for low‐density parasite infections which do not result in ‘soft flesh’ Giulietti et al. (2022).

Fish were stored at approximately 10°C for 48 h, then examined for signs of ‘soft flesh’, following guidelines provided by Giulietti et al. (2022, 2024b). Each fish was manually tested for muscle softening, and those showing any signs were dissected along the longitudinal side for visual inspection of the myomere structure, as described by Levsen, Jørgensen, and Mo (2008). If ‘soft flesh’ was observed, samples from the soft areas were prepared on glass slides, moistened with saline, and minced using a scalpel (St‐Hilaire et al. 1997a). The slides were examined under a brightfield microscope (up to 400× magnification) for the presence of myxospores. Muscle tissue from sardines suspected of ‘soft flesh’ was collected, stored in vials at −20°C and sent frozen to the Institute of Marine Research in Bergen, Norway, for molecular confirmation of *K. thyrsites* infection.

Additionally, muscle tissue was collected from a subsample of 70 (70/670) randomly selected sardine that did not exhibit visible ‘soft flesh’ from the August 2023 batch. These tissue samples were stored at −20°C and sent frozen to the Institute of Marine Research for further molecular analysis. All muscle samples received in the laboratory were screened for *K. thyrsites* infection using qPCR targeting the 18S rRNA gene. Genomic DNA was extracted from 60 mg of homogenate using the DNeasy Blood and Tissue Kit (Qiagen) following the manufacturer's instructions. DNA concentration was measured with a Qubit 2.0 Fluorometer (Thermo Fisher Scientific) and normalised to 10.0 ng/μl. *K. thyrsites* qPCR targeting an 82 bp SSU rDNA fragment (Funk et al. 2007) was performed as described by Giulietti et al. (2022), with DNA from previously examined infected and uninfected sardine tissues (Giulietti et al. 2024b) serving as positive and negative controls.

## 3 Results

The fish sampled in August 2023 had a mean total length of 131.5 mm (± 7.08 mm SD) and a mean total weight of 18 g (± 31.69 g SD), while those sampled in October 2024 had a mean total length of 137.3 mm (± 12.04 mm SD) and a mean total weight of 19 g (± 4.95 g SD). These measurements suggest that the samples predominantly consisted of mature individuals, based on the length at first maturity reported by Basilone et al. (2021). In the first batch of 670 sardines, two individuals displayed mild muscle softening indicative of the ‘soft flesh’ condition. However, microscopic examination of the affected tissue did not reveal myxospores and qPCR analysis resulted negative. qPCR analysis of muscle samples from the 70 randomly selected sardines without visible ‘soft flesh’ did not detect *K. thyrsites* infection.

## 4 Discussion

*Kudoa thyrsites* infection has been documented in European sardines from the NE Atlantic, where it is associated with the ‘soft flesh’ condition (Cruz, Silva, and Saraiva 2011; Iglesias et al. 2022; Giulietti et al. 2024b; Gilman and Eiras 1998). However, data from the Mediterranean remain scarce. This study represents the first investigation of *K. thyrsites* in sardines from the Tyrrhenian Sea, central Mediterranean.

Since ‘soft flesh’ occurs only in heavily parasitised individuals, its presence serves as an initial indicator of *K. thyrsites* infection, while molecular techniques are necessary for detecting low‐density infections. In our study, visual inspection of 1200 sardines and qPCR screening of a randomly selected subsample of 70 sardines did not detect *K. thyrsites*. None of the examined sardines exhibited ‘soft flesh’ consistent with *K. thyrsites* infection. Two individuals showed mild muscle softening, but microscopy and qPCR results were negative, suggesting postharvest handling as the likely cause (Giulietti et al. 2022).

While the absence of visibly affected fish does not confirm the parasite's true absence, the negative qPCR results further support a lack of infection in the sampled population. Negative findings in epidemiological studies must be interpreted cautiously, as undetected infections could result from low parasite prevalence, seasonal variability, or methodological constraints. Given previous reports of *K. thyrsites*‐induced myoliquefaction in NE Atlantic sardines from Morocco, Portugal, and the Cantabrian Sea (Cruz, Silva, and Saraiva 2011; Iglesias et al. 2022; Giulietti et al. 2024b; Gilman and Eiras 1998), the lack of infection recorded in the sample from the Tyrrhenian Sea is unexpected. However, *K. thyrsites* does not always induce muscle liquefaction, particularly at low parasite densities (St‐Hilaire et al. 1997b; Giulietti et al. 2022).

This suggests that *K. thyrsites* may be either present at very low prevalence and density—insufficient to induce soft flesh and with a prevalence so low that it was not detected in the genetic subsample analysed—or entirely absent due to ecological constraints or host unavailability in the central Tyrrhenian Sea. Reports of *K. thyrsites* in the Mediterranean are primarily from the westernmost regions, such as the Alboran Sea, or in highly migratory species like swordfish and Atlantic bonito (Giulietti et al. 2019; Panebianco et al. 2024; Gaglio et al. 2010). The Alboran Sea, where *K. thyrsites* has been documented in silver scabbardfish, represents an ecologically distinct area where Atlantic and Mediterranean waters mix, creating conditions more similar to the Atlantic ecosystem (Tintore et al. 1988). In contrast, the central Mediterranean may lack suitable environmental conditions, particularly regarding the presence of its likely annelid hosts (Eszterbauer et al. 2015; Rangel et al. 2016).

Another possibility is that *K. thyrsites* was absent in the specific subsample analysed. The sample size was appropriate for detecting infections at moderate to high prevalence, but detection at very low densities may require a larger sample.

## 5 Conclusion

*K. thyrsites* is well documented in sardines from the NE Atlantic, but this study found no evidence of infection in sardines from the central Mediterranean, specifically the Tyrrhenian Sea. Neither visual inspection nor qPCR detected *K. thyrsites*, suggesting that the parasite may be present at very low prevalence and density—insufficient to cause soft flesh—or entirely absent. Its absence or low prevalence in this population may be influenced by ecological constraints, including abiotic conditions and the availability of suitable hosts, or sample size limitations.

## Author Contributions

**Paolo Cipriani:** conceptualization, investigation, writing – original draft, visualization, writing – review and editing, supervision. **Marialetizia Palomba:** investigation, writing – review and editing, resources. **Luca Ruggiero:** resources, writing – review and editing. **Stig Mæle:** methodology, formal analysis. **Lucilla Giulietti:** conceptualization, writing – original draft, investigation, methodology, writing – review and editing, data curation, supervision.

## Conflicts of Interest

The authors declare no conflicts of interest.

## Data Availability

The data that support the findings of this study are available from the corresponding author upon reasonable request.
